# A decade of HAART in Latin America: Long term outcomes among the first wave of HIV patients to receive combination therapy

**DOI:** 10.1371/journal.pone.0179769

**Published:** 2017-06-26

**Authors:** Marcelo J. Wolff, Mark J. Giganti, Claudia P. Cortes, Pedro Cahn, Beatriz Grinsztejn, Jean W. Pape, Denis Padgett, Juan Sierra-Madero, Eduardo Gotuzzo, Stephany N. Duda, Catherine C. McGowan, Bryan E. Shepherd, for the Caribbean, Central and South America Network for HIV Epidemiology

**Affiliations:** 1 Fundación Arriarán, Facultad de Medicina, Universidad de Chile, Santiago, Chile; 2 Department of Biostatistics, Vanderbilt University School of Medicine, Nashville, TN, United States of America; 3 Fundación Huésped, Buenos Aires, Argentina; 4 Instituto Nacional de Infectologia Evandro Chagas Fiocruz, Rio de Janeiro, Brazil; 5 Le Groupe Haïtien d'Etude du Sarcome de Kaposi et des Infections Opportunistes, Port-au-Prince, Haiti; 6 Instituto Hondureño de Seguridad Social Hospital de Especialidades, Tegucigalpa, Honduras; 7 Department of Infectious Diseases, Instituto Nacional de Ciencias Médicas y Nutrición Salvador Zubirán, Talplan, Mexico; 8 Universidad Peruana Cayetano Heredia, Hospital Nacional Cayetano Heredia, Lima, Perú; 9 Department of Biomedical Informatics, Vanderbilt University School of Medicine, Nashville, TN, United States of America; 10 Department of Medicine, Vanderbilt University School of Medicine, Nashville, TN, United States of America; Katholieke Universiteit Leuven Rega Institute for Medical Research, BELGIUM

## Abstract

**Background:**

In Latin America, the first wave of HIV-infected patients initiated highly active antiretroviral therapy (HAART) 10 or more years ago. Characterizing their treatment experience and corresponding outcomes across a decade of HAART may yield insights relevant to the ongoing care of such patients and those initiating HAART more recently in similar clinical settings.

**Methods:**

This retrospective study included adults initiating HAART before 2004 at 8 sites in Argentina, Brazil, Chile, Haiti, Honduras, and Mexico. Patient status (in care, dead, or lost to follow-up [LTFU]) was assessed at 6-month intervals for 10 years, along with CD4 count and HIV-1 viral load (VL) for patients in care.

**Results:**

4,975 patients (66% male) started HAART prior to 2004; 45% were not antiretroviral-naïve. At 1, 5, and 10 years, rates of mortality were 4.2%, 9.0%, and 13.6% respectively. LTFU rates for the same periods were 2.4%, 10.9%, and 24.2%. Among patients remaining in care at 10 years, 84.4% were estimated to have VL≤400 copies/mL (Haiti excluded) and median baseline CD4 increased from 158 to 525 cells/mm^3^. Only 11.4% of all patients remained on their first regimen, 12.6% were on their second, 11.5% were on their third, and 23.0% were on their fourth or subsequent regimen. Outcomes were generally better for patients who were not antiretroviral-naïve, except for viral suppression. Heterogeneity among sites was substantial.

**Conclusions:**

Despite advanced disease and predominant use of older antiretrovirals, a large percentage of early HAART initiators in this Latin American cohort were alive and in care with sustained virologic suppression and progressive immune recovery after 10 years.

## Introduction

Expanded access to highly active antiretroviral therapy (HAART) in Latin America began in the late 1990s and accelerated during the early 2000s [1,2], replacing single or combined partially effective antiretroviral drugs and providing improved coverage. Most HAART initiators in these early years were in advanced disease stages, received regimens now considered outdated, and were poorly monitored by current standards [2]. Since this time, the region has developed better health infrastructure and a more skilled healthcare workforce. The World Health Organization (WHO) and national guidelines began recommending earlier treatment initiation of newer, safer, and more effective therapies [3–5] and are now recommending treatment for everyone, independent of clinical and laboratory status [6–8].

Nonetheless, there are still many regions in resource-limited countries where ART programs do not yet cover significant proportions of the infected population [2,9]. Many patients recently enrolled in care have advanced disease due to late diagnosis and/or poor linkage to care [10], and many of the antiretrovirals prescribed are the same used in the early years of HAART. Many persons living with HIV are yet to be diagnosed [2]. Hence, in many ways the situation for current HAART initiators in resource-limited countries largely mirrors that of early initiators in Latin America and elsewhere. The outcomes of those “HAART veterans” from the first wave of expanded access programs of HAART in Latin America have relevance not only for that population, but also for the growing cohort of patients receiving life-long HIV treatment.

In this study we evaluate the HIV treatment experience during the first ten years of therapy for patients who initiated antiretrovirals prior to 2004 during the first wave of HAART expansion in Latin America. We examine patient statuses/changes at six-month intervals over a decade of treatment, including viral suppression, CD4 recovery, regimen changes, loss to follow-up, and mortality. Using data from six countries with clinics that participate in a multi-centre cohort of HIV-infected adults from the Caribbean, Central and South America [11], we provide generalizable results that are useful in characterizing patient care over time in the region and that may be useful for understanding long-term patient outcomes under suboptimal treatment conditions elsewhere.

## Methods

This study included adult patients (≥ 18 years) who initiated HAART prior to 2004 at eight sites in the Caribbean, Central and South America Network for HIV Epidemiology (CCASAnet, www.ccasanet.org) [11]. Data included in this study came from the following sites: Hospital Fernández and Centro Médico Huésped in Buenos Aires, Argentina (HF/CMH-Argentina); Instituto Nacional de Infectologia Evandro Chagas in Rio de Janeiro, Brazil (INI-Brazil); Fundación Arriarán in Santiago, Chile (FA-Chile); Le Groupe Haitien d’Etude du Sarcome de Kaposi et des Infections Opportunistes in Port-au-Prince, Haiti (GHESKIO-Haiti); Instituto Hondureño de Seguridad Social and Hospital Escuela in Tegucigalpa, Honduras (IHSS/HE-Honduras); and Instituto Nacional de CienciasMédicas y Nutrición Salvador Zubirán in Mexico City, Mexico (INCMNSZ-Mexico).

Clinical, laboratory, and demographic data were collected at each site, de-identified, and securely uploaded to the CCASAnet Data Coordinating Centre at Vanderbilt University (VDCC) in Nashville, TN, USA, for data processing and merging. The VDCC checked data for internal consistency and performed on-site source document audits to verify the accuracy and completeness of the study data.

Institutional ethics review boards from all sites and the coordinating center at Vanderbilt University reviewed and approved the project (Comité de Bioética Fundación Huésped; Comité CEP IPEC del Instituto Nacional de Infectología Evandro Chagas; Comité de Ética Servicio de Salud Metropolitano Central-Chile; Comite des Droits Humains-Gheskio; Unidad de Investigación Científica-Universidad Nacional Autónoma de Honduras; Comité de Ética del Instituto Nacional de Ciencias Médicas y Nutrición Salvador Zubiran; Vanderbilt University Health Science Committee).

Patient data for the ten years following HAART initiation were included. A patient was classified as lost to follow-up (LTFU) if his/her last clinical visit occurred more than 90 days prior to the 10-year anniversary of HAART initiation. In these cases, the last visit date was used as the date of loss to follow-up. Patient death was ascertained using linkages with affiliated hospitals and notification by family members after a telephone call to the home due to patients missing a visit; in FA-Chile and INCMNSZ-Mexico, study staff additionally cross-checked LTFU subjects in national death registry databases.

HAART was defined as protease inhibitor (PI)-based (one ritonavir-boosted or unboosted PI plus two nucleoside reverse transcriptase inhibitors [NRTI]), non-nucleoside reverse transcriptase inhibitor (NNRTI)-based (one NNRTI plus two NRTIs), or other combinations (including triple NRTI regimens and all other regimens containing at least three drugs). Baseline HIV-1 viral load (VL) and CD4 count (CD4) were defined for each patient as the measurement closest to HAART initiation, though no more than 180 days before or 7 days after. Viral load and CD4 values used for each six month interval over the ten-year follow-up period were those measurements taken closest to the 6-month date within 90 days. If no measurement was taken within this window, VL and/or CD4 were classified as missing. During the ten-year follow-up period, the lower level detection limits for VL measurements varied within and among sites; to improve comparability, we categorized measurements as ≤400, 401–1000, or >1000 copies/mL.

Figures were constructed to show the cumulative proportion of patients dead or LTFU at six-month intervals for 10 years after starting HAART. Among patients in care (those not dead or LTFU), the proportion in each viral load category and the median CD4 and interquartile range (IQR) every 6 months were computed. To account for missing VL and CD4 among patients in care, observed values were weighted by the inverse probability of being missing. The probability of being missing was estimated using a logistic regression with the following covariates: age, site, sex, AIDS at HAART initiation, year of initiation, probable route of infection, initial HAART regimen, and prior ART use. Since VLs were not measured at GHESKIO-Haiti, data from this site was not used for the inverse probability weighted VL estimates. The percentages of patients in care with an observed VL and CD4 measurement at various time points are reported. Continuous and categorical variables were compared between patients with and without laboratory measurements using Wilcoxon rank sum and chi-square tests, respectively.

In addition to the proportion dead or LTFU, and VL and CD4 determinations, the proportion of patients on their first, second, third, or fourth or more regimen since HAART initiation at each day over the follow-up period was computed. Patients were classified as being on their first HAART regimen at time 0, including those with prior use of non-HAART antiretrovirals. This classification was carried forward until 1) at least one drug was changed in the regimen; 2) a documented ART interruption for more than 14 days occurred while the patient remained in care; 3) patient was classified as LTFU; or 4) patient died. We did not restrict our cohort to antiretroviral-naive patients and previous ART use was not characterized; thus, it is possible that different regimens may have been used prior to HAART. Sub-group analyses were also performed according to ART exposure status at HAART initiation.

Analyses were performed using R version 3.2.0 (r-project.org). Analysis scripts are posted at http://biostat.mc.vanderbilt.edu/ArchivedAnalyses.

## Results

A total of 4,975 patients met inclusion criteria: 1724 from HF/CMH-Argentina, 1374 from INI-Brazil, 701 from FA-Chile, 803 from GHESKIO-Haiti, 220 from IHSS/HE-Honduras, and 153 from INCMNSZ-Mexico. Table 1 shows the characteristics of patients at HAART initiation at each site and combined.

**Table 1:** Demographic and physiological characteristics for patients initiating antiretroviral therapy across six Caribbean, Central and South America sites (N = 4975).

	HF/CMH-Argentina	INI-Brazil	FA-Chile	GHESKIO-Haiti	IHSS/HE-Honduras	INCMNSZ-Mexico	Overall
	N = 1724	N = 1374	N = 701	N = 803	N = 220	N = 153	N = 4975
Age, years	33 (29–39)	36 (30–42)	35 (31–41)	39 (33–44.5)	34 (29–39)	34 (28–40)	35 (30–41)
Sex							
Female	487(28%)	489(36%)	110(16%)	455(57%)	117(53%)	26(17%)	1684(34%)
Male	1237(72%)	885(64%)	591(84%)	348(43%)	103(47%)	127(83%)	3291(66%)
Route of infection							
Heterosexual	565(33%)	628(46%)	210(30%)	0(0%)	123(56%)	55(36%)	1581(32%)
MSM	409(24%)	465(34%)	479(68%)	0(0%)	6(3%)	93(61%)	1452(29%)
IDU	178(10%)	39(3%)	5(1%)	0(0%)	1(0%)	4(3%)	227(5%)
Other	15(1%)	38(3%)	6(1%)	0(0%)	1(0%)	1(1%)	61(1%)
Unknown	557(32%)	204(15%)	1(0%)	803(100%)	89(40%)	0(0%)	1654(33%)
Clinical stage							
AIDS	355(21%)	78(6%)	202(29%)	304(38%)	116(53%)	79(52%)	1134(23%)
not AIDS	344(20%)	770(56%)	209(30%)	498(62%)	96(44%)	51(33%)	1968(40%)
Missing	1025(59%)	526(38%)	290(41%)	1(0%)	8(4%)	23(15%)	1873(38%)
Baseline CD4 count, cells/mL**	142 (48–265)	195 (81–325)	120 (39–219)	146 (68–219)	96 (44–198)	114 (36–228)	154 (60–258)
Missing	988(57%)	525(38%)	445(63%)	125(16%)	68(31%)	33(22%)	2184(44%)
Baseline viral load (log10)**	5.0 (4.4–5.5)	4.7 (4.0–5.3)	5.0 (4.5–5.5)	NA	5.0 (4.7–5.1)	4.9 (4.8–4.9)	4.9 (4.3–5.4)
Baseline viral load (undetectable)**							
Yes	48(3%)	37(3%)	5(1%)	0(0%)	1(0%)	1(1%)	92(2%)
No	647(38%)	661(48%)	424(60%)	0(0%)	36(16%)	110(72%)	1878(38%)
Missing	1029(60%)	676(49%)	272(39%)	803(100%)	183(83%)	42(27%)	3005(60%)
Initial regimen							
NNRTI	723(42%)	479(35%)	467(67%)	717(89%)	202(92%)	105(69%)	2693(54%)
Boosted PI	337(20%)	122(9%)	20(3%)	0(0%)	1(0%)	27(18%)	507(10%)
Unboosted PI	518(30%)	728(53%)	187(27%)	13(2%)	16(7%)	16(10%)	1478(30%)
3 NRTI	128(7%)	15(1%)	13(2%)	73(9%)	1(0%)	5(3%)	235(5%)
Other	18(1%)	30(2%)	14(2%)	0(0%)	0(0%)	0(0%)	62(1%)
Initiation Year							
1996	54(3%)	113(8%)	4(1%)	0(0%)	1(0%)	0(0%)	172(3%)
1997	141(8%)	237(17%)	9(1%)	0(0%)	2(1%)	0(0%)	389(8%)
1998	180(10%)	212(15%)	23(3%)	0(0%)	2(1%)	0(0%)	417(8%)
1999	188(11%)	156(11%)	114(16%)	0(0%)	1(0%)	0(0%)	459(9%)
2000	267(15%)	186(14%)	86(12%)	0(0%)	1(0%)	0(0%)	540(11%)
2001	334(19%)	199(14%)	122(17%)	1(0%)	6(3%)	5(3%)	667(13%)
2002	266(15%)	144(10%)	205(29%)	0(0%)	43(20%)	63(41%)	721(14%)
2003	294(17%)	127(9%)	138(20%)	802(100%)	164(75%)	85(56%)	1610(32%)
ART naive							
Yes	750(44%)	390(28%)	524(75%)	703(88%)	209(95%)	151(99%)	2727(55%)
No	973(56%)	984(72%)	177(25%)	99(12%)	11(5%)	2(1%)	2246(45%)
Unconfirmed	1(0%)	0(0%)	0(0%)	1(0%)	0(0%)	0(0%)	2(0%)

** Median baseline CD4 count and viral load measurements reported in this table are calculated using observed values only.

**Fig 1:**
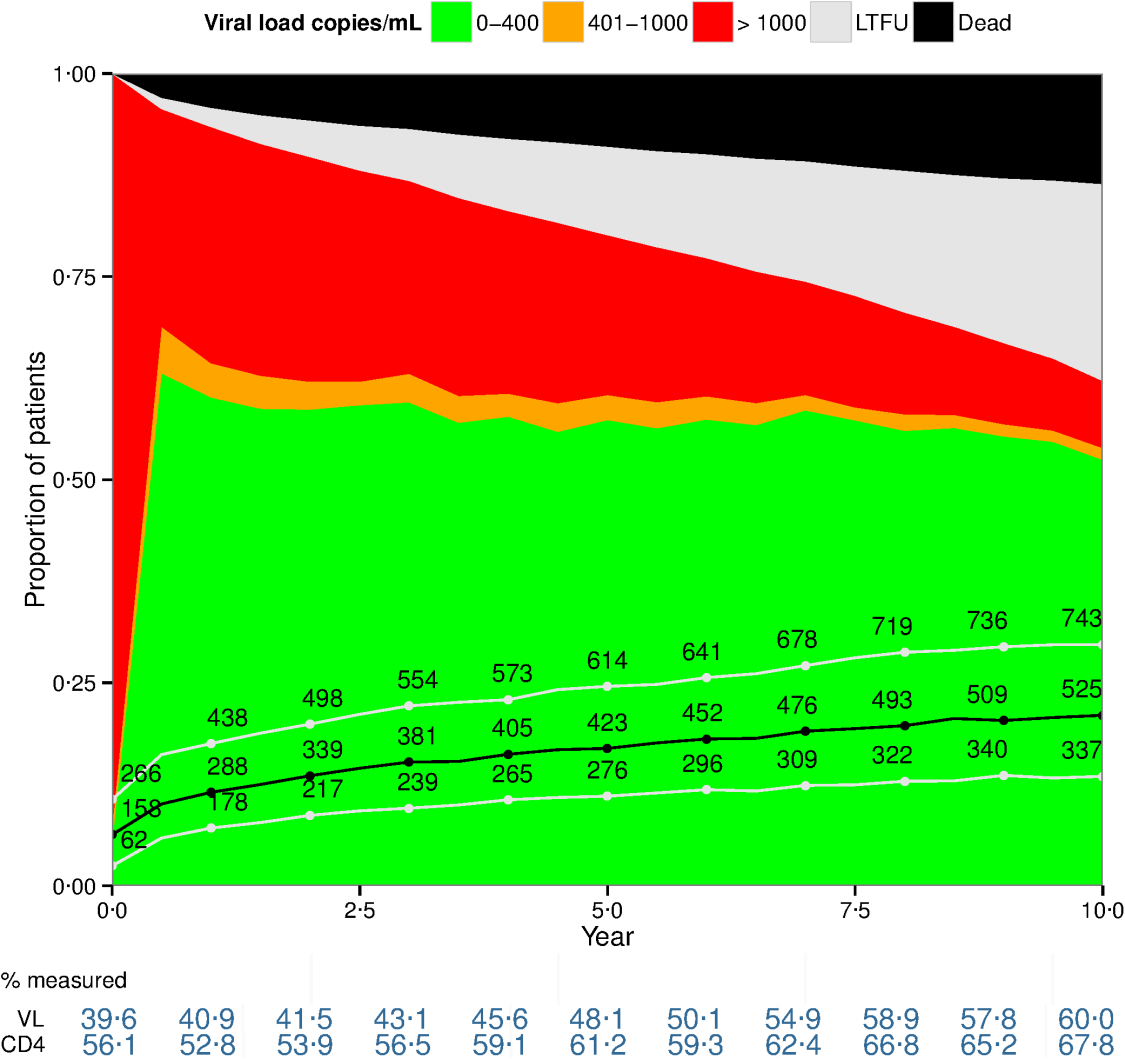
Clinical outcomes (death and lost to follow-up [LTFU]) during the first ten years of HAART among all patients who initiated HAART prior to 2004 at six CCASAnet sites (n = 4975). Median (black line) as well as 25^th^ and 75^th^ percentile CD4 counts (white lines) are also displayed. The percentage of active patients with a measured viral load and CD4 is given at the bottom of the figure. The proportions for viral load categories are based on the relative frequency of each category among active patients with a measurement during the six-month period. Haiti site didn´t measure viral load.

Overall, the majority of HAART initiators were male (66%); only GHESKIO-Haiti (57%) and IHSS/HE-Honduras (53%) were predominately female. The median age at HAART initiation was 35 years. Among patients with a laboratory measurement at baseline, the median CD4 at HAART initiation was 154 cells/mm^3^ (IQR: 60–258), ranging from 96 cells/mm^3^ in IHSS/HE-Honduras to 195 cells/mm^3^ in INI-Brazil. A total of 23% had clinical AIDS; however, AIDS status was unknown for 38% of patients. Whereas HF/CMH-Argentina, INI-Brazil, and FA-Chile had substantial proportions of patients initiating HAART prior to 2000, nearly all patients from GHESKIO-Haiti, IHSS/HE-Honduras, and INCMNSZ-Mexico initiated HAART in 2002 or 2003. Almost half (45%) of HAART initiators had exposure to antiretrovirals. Fig 1 (combined cohort) shows the rates of death, LTFU, retention in care, and viral suppression, as well as degree of immune recovery over the first ten years of HAART.

At 1, 3, 5, 7 and 10 years, the overall percentages of patients who had died were 4.2%, 6.8%, 9.0%, 10.8%, and 13.6% respectively. The percentages of patients who were LTFU at the same time points were 2.4%, 6.4%, 10.9%, 14.8%, and 24.2% respectively.

Among patients in care, the estimated median CD4 increased over the 10-year period, from 158 to 525 cells/mm^3^ at 10 years. Overall, 53.6% of patients had reached a CD4 ≥500 cells/mm^3^, varying from 36.8% in IHSS/HE-Honduras to 67.5% in GHESKIO-Haiti. Sixty-two percent of patients remained in care at 10 years after HAART initiation. Excluding Haiti, 84.4% of retained patients from the other sites had virologic suppression (VL< 400 copies/mL) at 10 years.

Results, however, were markedly heterogeneous across cohorts (S1 Fig). Both early and long-term mortality rates varied across sites. Early mortality was especially pronounced in GHESKIO-Haiti and IHSS/HE-Honduras. After 10 years, 3.5% of patients from HF/CMH-Argentina were known to have deceased compared with 19.5%, 21.6%, and 23.3% from IHSS/HE-Honduras, INI-Brazil, and GHESKIO-Haiti, respectively. In contrast, 44.4% of patients from HF/CMH-Argentina were classified as LTFU at 10 years compared to 4.9% from INI-Brazil. The proportion of patients who were in care with detectable viral load (VL>400 copies/mL) was substantially higher for most of the follow-up period at INI-Brazil.

Substantial proportions of patients were missing VL and CD4 measurements, particularly in the earlier years. Only forty percent, 48%, and 60% of active patients had a viral load measurement at baseline, year 5, and year 10, respectively. Excluding GHESKIO-Haiti, 47%, 55%, and 69% of patients in care had a VL measurement at baseline, year 5, and year 10, respectively. Availability of VL measurements varied across sites, ranging from 16.8% at IHSS/HE-Honduras at baseline to 86.7% at INCMNSZ-Mexico at year 10. A comparison of patients with and without viral load measurements one year after HAART initiation according to site is included in the Supplemental Material (S1, S2 and S3 Tables).

**Fig 2:**
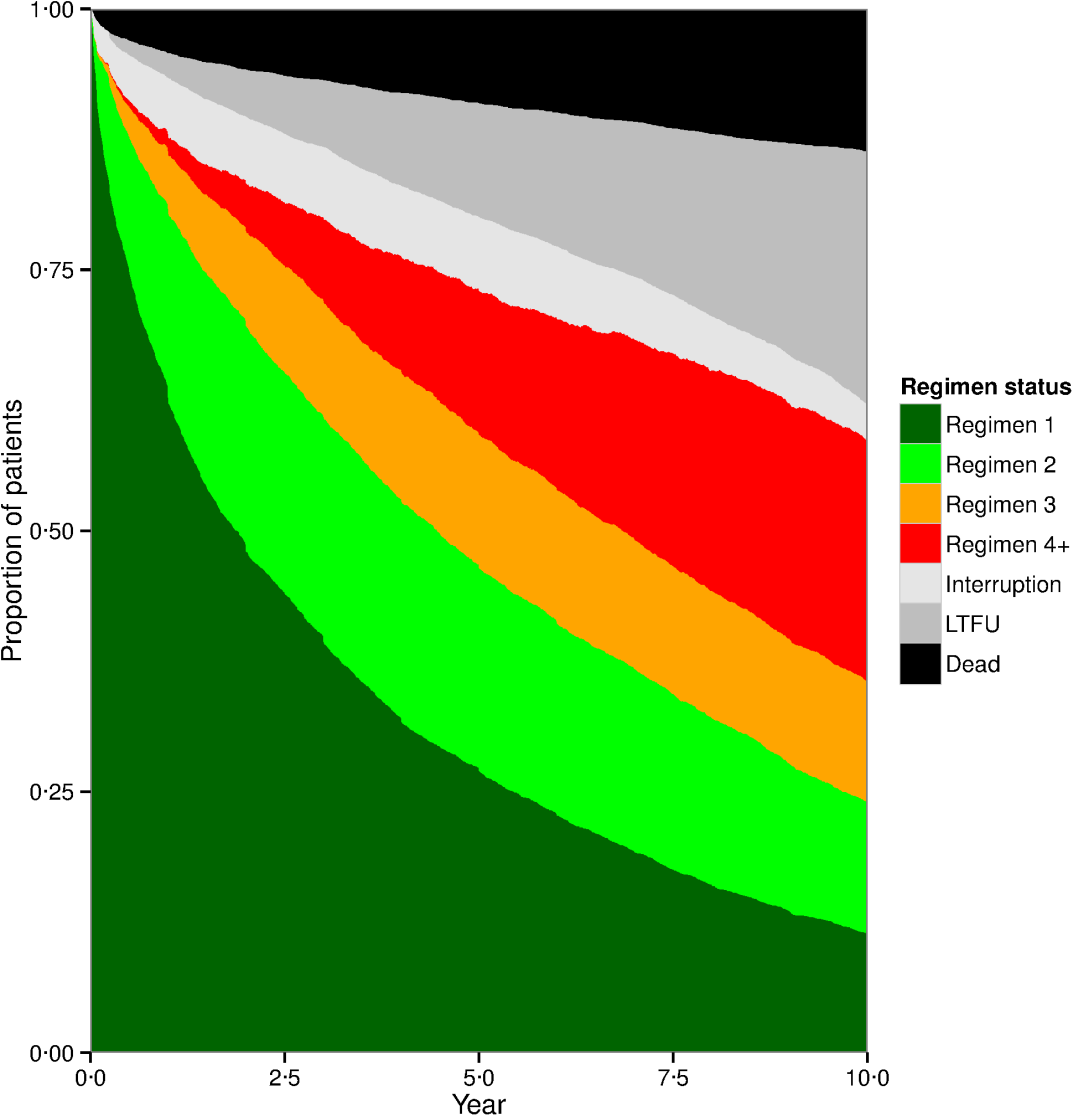
Drug regimen status during the first ten years of HAART among all patients who initiated treatment prior to 2004 (n = 4,975).

Fig 2 (combined cohort) shows the proportion of patients on their first, second, third, or fourth or subsequent regimen for the first 10 years following HAART initiation. The proportion not receiving antiretrovirals (i.e., drug interruption, LTFU, or dead) is also shown.

After 10 years, only 11.4% of patients remained in care and on their first HAART regimen, 12.6% were on their second, 11.5% were on their third, and 23.0% were on their fourth or subsequent regimen. Periods of time with no antiretroviral use were not uncommon, particularly during years 1–7. Site-specific figures are given in the Supplemental Material (S2 Fig).

Among patients on HAART, the percentage of patients receiving a non-nucleoside reverse transcriptase inhibitor (NNRTI)-based regimen changed only modestly from baseline (54.1%) to 5 years (59.2%) and 10 years (50.4%). Meanwhile, the percentage of patients receiving a boosted protease inhibitor (PI)-based regimen increased between baseline (10.2%), 5 years (20.9%), and 10 years (40.8%).

**Fig 3:**
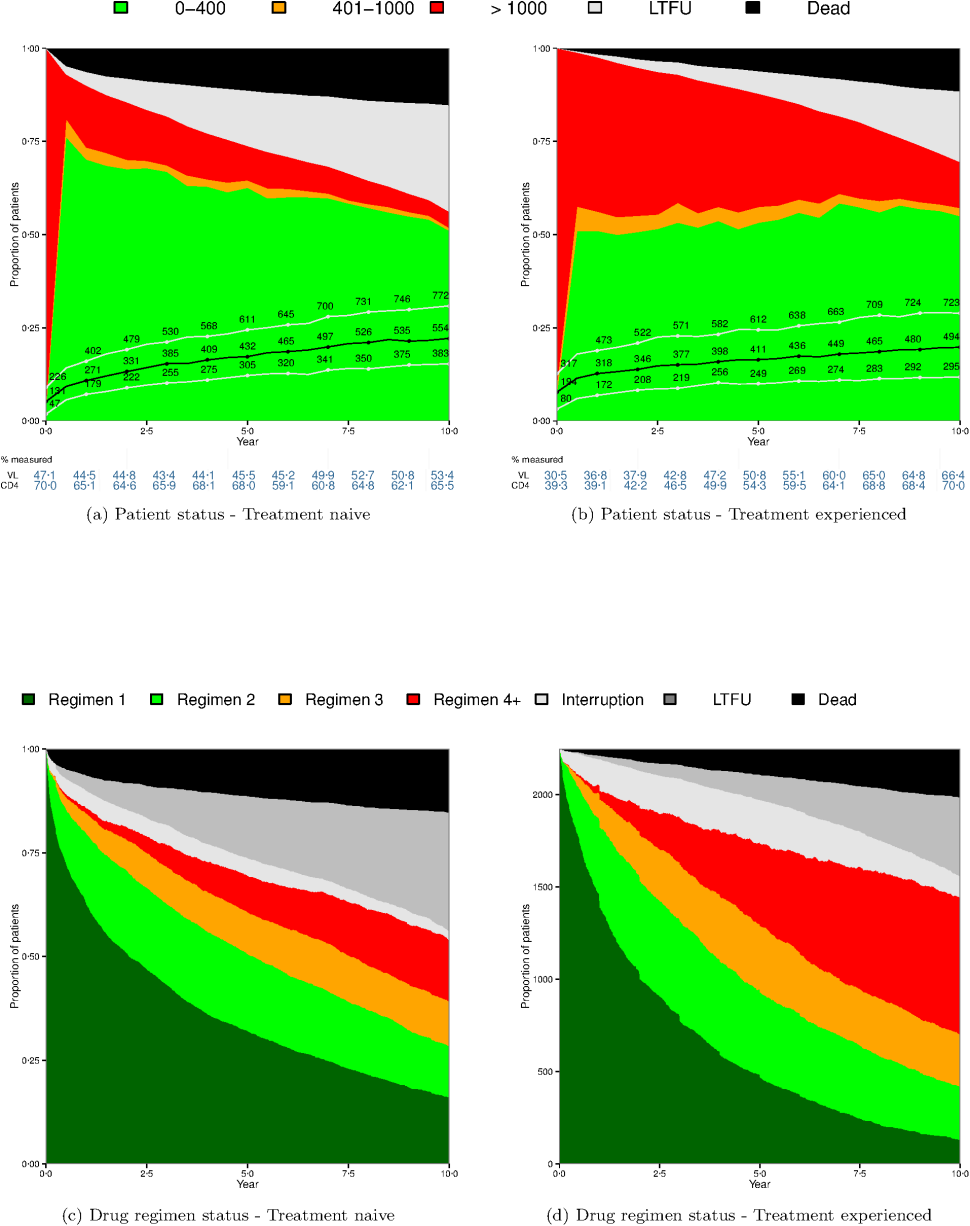
**Viral load and CD4 values and drug regimen status during the first ten years of HAART among 2727 ART-naïve (panels a and c) and 2248 treatment-experienced (panels b and d) patients who initiated HAART prior to 2004 at six CCASAnet sites**. See legend for Fig 1 for more details.

Analyses were repeated differentiating patients who were antiretroviral-naive at HAART initiation (n = 2,727) from those who were treatment-experienced (n = 2,248) (Fig 3A and 3B).

The death rate was higher for treatment-naïve patients throughout the observation period (6.3% and 15.3% versus 1.6% and 11.6% at years 1 and 10, respectively, p<0.001). Similarly, LTFU was greater in the treatment-naive population (3.7% and 28.6% versus 0.8% and 19% in treatment-experienced patients at years 1 and 10 respectively, p<0.001). The frequency of viral suppression was higher in treatment-naïve patients in care (VL<400 copies/mL in 78.0% at year 1 and 91.0% at year 10) than in treatment-experienced patients (52.3% and 79.1% at years 1 and 10, respectively). Median CD4 for the treatment-naïve cohort increased from 131cells/mm^3^ at baseline to 554 cells/mm^3^ at 10 years; for treatment-experienced patients, median CD4 increased from 194 at baseline to 494 at 10 years. These differences were quite consistent for all sites.

Fig 3C and 3D show the proportion of treatment-naïve and treatment-experienced patients on their first, second, third, or fourth or subsequent regimen for the first 10 years following HAART initiation. The former group had lower rates of regimen change compared with treatment-experienced patients, and first regimen was maintained nearly 3 times more frequently at year 10 (16.1% vs 5.8%).

## Discussion

The aim of this study was to evaluate the treatment experience over ten years for patients initiating HAART during the early years of its availability in Latin America. Despite large heterogeneity between sites, corresponding with considerable diversity across the region, we found that almost two-thirds of patients were alive and in care after 10 years. Documented mortality was 14% and LTFU 24%. Among those in care at sites where VL was performed (all but GHESKIO-Haiti), 84% had an undetectable viral load, and median CD4 had improved remarkably, from 158 cells/mm^3^ at HAART initiation to 525 cells/mm^3^ at the end of 10 years. This can be considered a significant achievement for the region, where ART programs were largely funded with their own, typically limited, resources. The population studied reflects the HIV epidemic in the region before 2004: predominantly males in their thirties (except in Haiti and Honduras, the two countries with the most economic hardship) with sexually-acquired infection, large proportions of men who have sex with men, low rates of intravenous drug use, and the majority with advanced disease.

High early mortality was observed, as has been documented previously in the region [12, 13] and in many other studies [14–16]; almost a third of deaths occurred in the first year and half occurred during the first 2 years, after which the rate of mortality was lower, except at the site in Brazil. Reported death and LTFU rates varied considerably across sites. The mortality rate at HF/CMH-Argentina is likely underreported, and probably hidden within the high rate of LTFU (44%) at that site. Investigators from HF/CMH-Argentina have indicated that those cases were mainly transfers to other sites due to health insurance issues or new clinic openings, although this has not been sufficiently documented.

For this study, we chose to include all HAART initiators, some of who were not antiretroviral-naïve at the time of HAART initiation or may have started treatment at other sites. We included these patients to reflect the reality of HIV care in our region during the first wave of HAART, and to evaluate the long-term impact of that experience. Although we presume that many, if not most, of the treatment-experienced patients had received mono- or dual- therapy previously, we cannot rule out that some may have started HAART regimens at other clinics prior to enrolment in care at CCASAnet sites. Our sensitivity analysis, including only patients who were ART-naïve at HAART initiation at a CCASAnet clinic, yielded similar, albeit slightly lower survival rates at 10 years. Although the negative impact of prior non-HAART use has been reported [17, 18], we believe that the slight decrease in survival seen in our study when limited to ART-naïve HAART initiators may reflect a survival bias: patients who started ART prior to entry into a CCASAnet clinic by definition had to survive long enough to start care at the clinic, and some early deaths were therefore unobserved. Nonetheless, some deleterious effects of prior antiretroviral exposure were observed: a lower rate of viral suppression, blunted CD4 response, and higher frequency of regimen change. Although all patients now initiate treatment with HAART regimens, use of less effective or outdated drug combinations still persists in some regions [2, 19] and may affect long-term outcomes as those seen here.

Laboratory monitoring was initially infrequent and irregular, reflecting the reality of that time and resources at the sites. Many patients started HAART without VL measurements, probably more so in cases of clinically advanced disease or very low CD4 in which treatment could not wait for VL determinations. A similar, but less severe, situation was seen with CD4. The percentage of patients with laboratory values steadily increased over time, implying improved availability of monitoring tools.

A substantial proportion of patients had undetectable VL since their early years of HAART and this proportion increased over time, with all the positive implications for their health and that of others (due to reduced infectivity). Similarly, immune recovery was substantial, more than tripling over 10 years. Although we did not study clinical outcomes other than death, the improvement in virologic and immunologic outcomes would predict lower risks of opportunistic infections [7, 20, 21].

Therapy was begun with NNRTI-based regimens in 54% of patients and only 10% initiated boosted PI regimens (although 30% started a regimen containing an unboosted PI). These regimens evolved slightly, mainly with more use of boosted PIs. Patients changed therapeutic regimens frequently, with a small minority (16% for treatment-naïve and 5.8% in treatment experienced patients) maintaining their original regimen after 10 years. Although we did not report the reasons for changes in this population, a previous CCASAnet study with a larger number of ART-naïve patients initiating therapy over a wider time span found that the primary reason for early change was toxicity (57%), whereas treatment failure accounted for early change in only 5% of patients [22]. Recent CCASAnet data has shown that 61% of those who changed their first regimen changed to a third by 5 years [23]. Similar findings have been reported elsewhere, even in populations initiating HAART in more recent years [24, 25]. Undoubtedly, in the present study, change of regimens may have been less frequent had less toxic drugs or more appropriate combinations been available or used.

The ten years of follow-up in this study represents “long-term” in HIV care in the current literature, but we acknowledge there is no consensus on what “long-term” means. It has varied from the early years of antiretroviral therapy when long-term was considered a couple of years [26,27] to the mid-2000s when long-term was considered longer periods (i.e., 4–7 years) [28–30]. While 10 years may not be truly long-term for a life-long therapy, there will always be a group of 10-year veterans of whatever treatment modality is in use at a given time whose experience will be useful for their peers and their caregivers. There are reports of ART treatment programs of 10–15 years [31–34], but none from Latin America and their actual median follow-up is typically much shorter and outcomes at longer periods are projections based on smaller numbers of patients. In this study, similar to recent reports from Baltimore [35] and Haiti [36], 10-year outcomes were from the complete population in “real life” situations, experiencing the evolving nature of HIV management over a decade.

In addition to those mentioned above, our study has several limitations, most due to the inherent weaknesses of a retrospective study. The participating cohorts, mostly from urban areas, may not fully represent the situation of their countries as a whole and did not include children. Our overall evaluation was based on simple aggregation of all patient records and did not adjust for certain clinics contributing larger numbers of patients. As results may be driven by underlying factors at those large sites, we recommend prudence in interpreting the overall findings as well as consideration for the site-specific analyses in the Supplemental Materials. Status of those lost to follow-up is not well known; they may have abandoned treatment, received care at other sites, or died. Missing laboratory values may not be well represented by those that were measured; thus true rates of undetectable viral load and CD4 may differ from those reported here. Finally, significant clinical information beyond death was not reported.

Nonetheless, this study represents the largest regional multi-site, long-term follow-up cohort from Latin America, a region in which data regarding the HIV epidemic are relatively scarce [37–40].

These results of patients who started treatment in the early years of HAART to present-day care are relevant in many ways. First, they show that in a region with a mostly moderate but heterogeneous epidemic that rolled out HIV treatment largely with their own resources, the majority of patients are still alive and in care with virologic suppression and greatly improved immune status after ten years. These patients, and the large number of similar ones who started HAART in subsequent years, still require proper care and may have special needs arising from drug and aging-related co-morbidities as well as antiretroviral restrictions due to prior use. Second, the experience of HAART veterans is not outdated. There remain millions of HIV-infected people in need of diagnosis of the infection and urgent care–primarily in resource-limited countries, including Latin America and the Caribbean, many or most of whom have advanced disease similar to the population of this study [2]. Despite WHO recommendations for treatment of everyone living with HIV regardless of CD4 count, the median baseline CD4 of those initiating treatment everywhere, especially in resource-limited countries, is far lower than the cut-off previously recommended [3], and contemporary antiretrovirals and monitoring tools are not yet universally available [41, 42]. The characterization of the first decade of HAART in our cohort will not only help these veteran patients, but also those hoping to reach contemporary long-term treatment goals to implement the best possible care.

## Supporting information

S1 Fig Site-specific patient outcomes during the first ten years of HAART among all patients who initiated HAART prior to 2004. Median (black line) as well as 25th percentile and 75th percentile CD4 (white lines) are also displayed. The percentage of active patients with a measured viral load and CD4 is given at the bottom of the figure.
(PDF)

S2 Fig Site-specific drug regimen status during the first ten years of HAART among all patients who initiated treatment prior to 2004. Patients were classified as lost to follow-up after their last documented visit date, regardless of the timing of the previous regimen dispensation.
(PDF)

S1 Table Demographic characteristics of patients who were active at one year and have a viral load measurement (n = 1899). (PDF)

S2 Table Demographic characteristics of patients who were active at one year but did not have a viral load measurement (n = 2072). (PDF)

S3 Table P-value comparison of demographic characteristics of patients who were active at one year and have a viral load measurement compared to those without a viral load measurement (n = 3971). P-values for continuous variables were calculated using a Wilcoxon Rank Sum test. P-values for categorical variables were calculated using a Chi-squared test.
(PDF)

## References

[1] Chequer P, Cuchí P, Mazin R, García Calleja JM. Access to antiretroviral treatment in Latin American countries and the Caribbean. AIDS. 2002;16 Suppl 3:S50–7. 12685925 10.1097/00002030-200212003-00008

[2] Pan American Health Organization Antiretroviral treatment in the spotlight: a public health analysis in Latin America and the Caribbean. Washington, D.C.: PAHO, © 2013.

[3] World Health Organization Consolidated guidelines on the use of antiretroviral drugs for treating and preventing HIV infection June 2013. http://www.who.int/hiv/pub/guidelines/arv2013/download/en/index.html (accessed December 13, 2015). 24716260

[4] British HIV Association guidelines for the treatment of HIV-1-positive adults with antiretroviral therapy 2012. HIV Medicine (2014), 15 (Suppl. 1), 1–85. 10.1111/j.1468-1293.2012.01029.x 22830364

[5] US Department of Health and Human Sciences (DHHS). Guidelines for the use of antiretroviral agents in HIV-1-infected adults and adolescents. February 2013. http://www.aidsinfo.nih.gov/guidelines (accessed December 13, 2015)

[6] World Health Organization Guideline on when to start antiretroviral therapy and on pre-exposure prophylaxis for HIV. September 2015. www.who.int/iris/bitstream/10665/186275/1/9789241509565_eng.pdf (accessed December 13, 2015) 26598776

[7] INSIGHT START Study Group, Lundgren JD, Babiker AG, Gordin F, Emery S, Grund B, Sharma S, et al Initiation of Antiretroviral Therapy in Early Asymptomatic HIV Infection. N Engl J Med. 2015;373(9):795–807 doi: 10.1056/NEJMoa1506816 26192873 10.1056/NEJMoa1506816 PMC4569751

[8] US Department of Health and Human Sciences (DHHS) Guidelines for the Use of Antiretroviral Agents in HIV-1-Infected Adults and Adolescents. May 2014. http://aidsinfo.nih.gov/guidelines (accessed December 13, 2015)

[9] UNAIDS. The Gap Report. 2014 http://www.unaids.org/sites/default/files/media_asset/UNAIDS_Gap_report_en.pdf (accessed December 13, 2015)

[10] Crabtree-Ramírez B, Caro-Vega Y, Shepherd BE, Wehbe F, Cesar C, Cortes C, et al, for the CCASAnet Team. Cross-sectional analysis of late HAART initiation in Latin America and the Caribbean: late testers and late presenters. PLoS One. 2011;6(5):e20272 doi: 10.1371/journal.pone.0020272 21637802 10.1371/journal.pone.0020272 PMC3102699

[11] McGowan CC, Cahn P, Gotuzzo E, Padgett D, Wolff M, Schechter M, et al Cohort Profile: Caribbean, Central and South America Network for HIV research (CCASAnet) collaboration within the International Epidemiologic Databases to Evaluate AIDS (IeDEA) programme. Int J Epidemiol 2007;36: 969–976 doi: 10.1093/ije/dym073 17846055 10.1093/ije/dym073

[12] Grinsztejn B, Veloso VG, Friedman RK, Moreira RI, Luz PM, Campos DP, et al Early mortality and cause of deaths in patients using HAART in Brazil and the United States. AIDS. 2009;23:2107–14. doi: 10.1097/QAD.0b013e32832ec494 19770698 10.1097/QAD.0b013e32832ec494 PMC3790467

[13] Tuboi SH, Schechter M, McGowan CC, Cesar C, Krolewiecki A, Cahn P et al Mortality during the first year of potent antiretroviral therapy in HIV-1-infected patients in 7 sites throughout Latin America and the Caribbean. J Acquir Immune Defic Syndr. 2009;51:615–23. doi: 10.1097/QAI.0b013e3181a44f0a 19430306 10.1097/QAI.0b013e3181a44f0a PMC2780368

[14] Lawn SD, Harries AD, Anglarete X, Myer L and, Wood R. Early mortality among adults accessing antiretroviral treatment programmes in sub-Saharan Africa. AIDS 2008, 22:1897–190 doi: 10.1097/QAD.0b013e32830007cd 18784453 10.1097/QAD.0b013e32830007cd PMC3816249

[15] Gupta A, Nadkarni G, Yang W-T, Chandrasekhar A, Gupte N, Bisson GP et al Early Mortality in Adults Initiating Antiretroviral Therapy (ART) in Low- and Middle-Income Countries (LMIC): A Systematic Review and Meta-Analysis. PLoS ONE 6(12): e28691 doi: 10.1371/journal.pone.0028691 22220193 10.1371/journal.pone.0028691 PMC3248405

[16] The Antiretroviral Therapy in Lower Income Countries (ART-LINC) Collaboration and ART Cohort Collaboration (ART-CC) groups. Mortality of HIV-1-infected patients in the first year of antiretroviral therapy: comparison between low-income and high-income countries. Lancet. 2006 (367):817.24 10.1016/S0140-6736(06)68337-2 16530575

[17] Ségéral O, Limsreng S, Nouhin J, Hak C, Ngin S, De Lavaissière M et al Short Communication: Three Years Follow-Up of First-Line Antiretroviral Therapy in Cambodia: Negative Impact of Prior Antiretroviral Treatment. AIDS Research and Human Retroviruses. 6 2011, 27(6): 597–603. doi: 10.1089/AID.2010.0125 21083413 10.1089/AID.2010.0125

[18] Ballif M, Ledergerber B, Battegay M, Cavassini M, Bernasconi E, Schmid P, et al; for the Swiss HIV Cohort Study. Impact of previous virological treatment failures and adherence on the outcome of antiretroviral therapy in 2007. PLoS One. 2009 12 14;4(12):e8275 doi: 10.1371/journal.pone.0008275 20011544 10.1371/journal.pone.0008275 PMC2789943

[19] World Health Organization. AIDS Medicines and diagnostic service. Antiretroviral Medicines in Low- and- Middle- Income Countries: Forecast of Global and Regional Demand for 2013–2016. March 2014. http://who.int/iris/bitstream/10665/111626/1/9789241507004_eng.pdf (accessed December 13, 2015)

[20] Mocroft A, Furrer HJ, Miro JM, Reiss P, Mussini C, Kirk O, et al, for the Opportunistic Infections Working Group on behalf of the Collaboration of Observational HIV Epidemiological Research Europe (COHERE) study in EuroCOORD. The incidence of AIDS-defining illnesses at a current CD4 count ≥ 200 cells/μL in the post-combination antiretroviral therapy era. Clin Infect Dis. 2013;57(7):1038–47 doi: 10.1093/cid/cit423 23921881 10.1093/cid/cit423

[21] Cohen MS, Chen YQ, McCauley M, Gamble T, Hosseinipour MC, Kumarasamy N, et al, for the HPTN 052 Study Team. Prevention of HIV-1 Infection with Early Antiretroviral Therapy. N Engl J Med 2011; 365:493–505 doi: 10.1056/NEJMoa1105243 21767103 10.1056/NEJMoa1105243 PMC3200068

[22] Cesar C, Shepherd BE, Krolewiecki A, Fink VI, Schechter M, Tuboi SH, et als, for The Caribbean, Central and South America Network for HIV Research (CCASAnet) Collaboration, of the International Epidemiologic Databases to Evaluate AIDS (IeDEA) Program. Rates and Reasons for Early Change of First HAART in HIV-1-Infected Patients in 7 Sites throughout the Caribbean and Latin America. PLoS ONE 6 1, 2010 5(6): e10490 doi: 10.1371/journal.pone.0010490 20531956 10.1371/journal.pone.0010490 PMC2879360

[23] Wolff M, Shepherd BE, Cortés CP, Rebeiro P, Cesar C, Wagner Cardoso S, et als; for The Caribbean, Central and South America Network for HIV Epidemiology (CCASAnet). Clinical and virologic outcomes after changes in first antiretroviral regimen at 7 sites in the Caribbean, Central and South America Network (CCASAnet). J Acquir Immune Defic Syndr. 2016;71 (1):102–110 doi: 10.1097/QAI.0000000000000817 26761273 10.1097/QAI.0000000000000817 PMC4712722

[24] Cicconi P, Cozzi-Lepri A, Castagna A, Trecarichi EM, Antinori A, Gatti F, et als, for the ICoNA Foundation Study Group. Collaborators). Insights into reasons for discontinuation according to year of starting first regimen of highly active antiretroviral therapy in a cohort of antiretroviral-naïve patients. HIV Med. 2010 2;(2):104–13 doi: 10.1111/j.1468-1293.2009.00750.x 19732176 10.1111/j.1468-1293.2009.00750.x

[25] Wright S, Boyd MA, Yunihastuti E, Law M, Sirisanthana T, Hoy J, et al Rates and factors associated with major modifications to first-line combination antiretroviral therapy: results from the Asia-Pacific region. PLoS ONE. 2013;8(6):e64902 doi: 10.1371/journal.pone.0064902 23840312 10.1371/journal.pone.0064902 PMC3696001

[26] Girard PM, Guiguet M, Bollens D, Goderel I, Meyohas MC, Lecomte I, et al, for Triest Cohort Investigators. Long-term outcome and treatment modifications in a prospective cohort of human immunodeficiency virus type 1-infected patients on triple-drug antiretroviral regimens. Clin Infect Dis. 2000;31(4):987–94 doi: 10.1086/318154 11049781 10.1086/318154

[27] Sabin CA, Fisher M, Churchill D, Pozniak A, Hay P, Easterbrook P, et al Long-term follow-up of antiretroviral-naive HIV-positive patients treated with nevirapine. Acquir Immune Defic Syndr. 2001 4 15;26(5):462–5. 10.1097/00126334-200104150-00009 11391166

[28] Hicks C, King MS, Gulick RM, White AC Jr, Eron JJ Jr, Kessler HA, et al Long-term safety and durable antiretroviral activity of lopinavir/ritonavir in treatment-naive patients: 4 year follow-up study. AIDS. 2004 3 26;18(5):775–9. 15075512 10.1097/00002030-200403260-00008

[29] García F, de Lazzari E, Plana M, Castro P, Mestre G, Nomdedeu M, et al Long-term CD4+ T-cell response to highly active antiretroviral therapy according to baseline CD4+ T-cell count. J Acquir Immune Defic Syndr. 2004;36(2):702–13. 15167289 10.1097/00126334-200406010-00007

[30] Lok JJ, Bosch RJ, Benson CA, Collier AC, Robbins GK, Shafer RW, et al for the ALLRT team. Long-term increase in CD4+ T-cell counts during combination antiretroviral therapy for HIV-1 infection. AIDS. 2010;24(12):1867–76 doi: 10.1097/QAD.0b013e32833adbcf 20467286 10.1097/QAD.0b013e32833adbcf PMC3018341

[31] Patel K, Hernán MA, Williams PL, Seeger JD, McIntosh K, Van Dyke RB, et al, for the Pediatric AIDS Clinical Trials Group 219/219C Study Team. Long-term effectiveness of highly active antiretroviral therapy on the survival of children and adolescents with HIV infection: a 10-year follow-up study. Clin Infect Dis. 2008;46(4):507–15. doi: 10.1086/526524 18199042 10.1086/526524

[32] Mouala C, Madec Y, Adam G, Courpotin C, Fikouma V, Gentilini M, et als. Ten years of commitment to persons living with HIV-AIDS: evaluation of the management in three ambulatory treatment centers of the French Red Cross in Africa. Sante.2008;18(2):89–95. doi: 10.1684/san.2008.0117 19188132 10.1684/san.2008.0117

[33] Grimsrud A, Balkan S, Casas EC, Lujan J, Van Cutsem G, Poulet E, et al Outcomes of antiretroviral therapy over a 10-year period of expansion: a multicohort analysis of African and Asian HIV programs. J Acquir Immune Defic Syndr. 2014 10 1;67(2):e55–66 doi: 10.1097/QAI.0000000000000268 24977472 10.1097/QAI.0000000000000268

[34] May MT, Vehreschild JJ, Trickey A, Obel N, Reiss P, Bonnet F, et al Mortality According to CD4 Count at Start of Combination Antiretroviral Therapy Among HIV-infected Patients Followed for up to 15 Years After Start of Treatment: Collaborative Cohort Study. Clin Infect Dis. 2016;62(12):1571–7. doi: 10.1093/cid/ciw183 27025828 10.1093/cid/ciw183 PMC4885653

[35] Lesko CR, Edwards JK, Moore RD, Lau B. A longitudinal, HIV care continuum: 10-year restricted mean time in each care continuum stage after enrollment in care, by history of injection drug use. AIDS. 2016 6 29. 10.1097/QAD.0000000000001183 PMC5063502 27314178

[36] Pierre S, Jannat-Khah D, Fitzgerald DW, Pape J, McNairy ML. 10-Year Survival of Patients with AIDS Receiving Antiretroviral Therapy in Haiti. New England Journal of Medicine. 2016 1 28;374(4):397–8. doi: 10.1056/NEJMc1508934 26816026 10.1056/NEJMc1508934 PMC4824285

[37] Koenig SP, Rodriguez LA, Bartholomew C, Edwards A, Carmichael TE, Barrow G, et al Long-term antiretroviral treatment outcomes in seven countries in the Caribbean. J Acquir Immune Defic Syndr. 2012;59:60–71. 10.1097/QAI.0b013e318245d3c1 PMC3299899 22240464

[38] Belloso WH, Orellana LC, Grinsztejn B, Madero JS, La Rosa A, Veloso VG, et al Analysis of serious non-AIDS events among HIV-infected adults at Latin American sites. HIV Med. 2010;11(9):554–64 doi: 10.1111/j.1468-1293.2010.00824.x 20345879 10.1111/j.1468-1293.2010.00824.x

[39] Stoszek SK, Duarte G, Hance LF, Pinto J, Gouvea MI, Cohen RA, et als, for the NISDI Perinatal/LILAC Study Group. Trends in the management and outcome of HIV-1-infected women and their infants in the NISDI Perinatal and LILAC cohorts, 2002–2009. Int J Gynaecol Obstet. 2013;122(1):37–43. doi: 10.1016/j.ijgo.2012.12.021 23566742 10.1016/j.ijgo.2012.12.021 PMC4880059

[40] Wolff MJ, Cortés CP, Shepherd BE, Beltrán CJ; Chilean AIDS Cohort Study Group. Long-term outcomes of a national expanded access program to antiretroviral therapy: the Chilean AIDS cohort. J Acquir Immune Defic Syndr. 2010;55:368–74. doi: 10.1097/QAI.0b013e3181eb4fb9 20683194 10.1097/QAI.0b013e3181eb4fb9

[41] Duda SN, Farr AM, Lindegren ML, Blevins M, Wester CW, Wools-Kaloustian K, et als for the International Epidemiologic Databases to Evaluate AIDS (IeDEA) Collaboration. Characteristics and comprehensiveness of adult HIV care and treatment programmes in Asia-Pacific, sub-Saharan Africa and the Americas: results of a site assessment conducted by the International epidemiologic Databases to Evaluate AIDS (IeDEA) Collaboration. J Int AIDS Soc. 2014;17:19045 doi: 10.7448/IAS.17.1.19045 25516092 10.7448/IAS.17.1.19045 PMC4268491

[42] Kumarasamy N, Krishnan S. Beyond first-line HIV treatment regimens: the current state of antiretroviral regimens, viral load monitoring, and resistance testing in resource-limited settings. Curr Opin HIV AIDS. 2013;8(6):586–90 doi: 10.1097/COH.0000000000000004 24100872 10.1097/COH.0000000000000004

