# Supplementary figures and images for: A decade of HAART in Latin America: Long term outcomes among the first wave of HIV patients to receive combination therapy
Source: PLoS One. 2017 Jun 26;12(6):e0179769. doi: 10.1371/journal.pone.0179769 (PMC5484471; doi:10.1371/journal.pone.0179769)

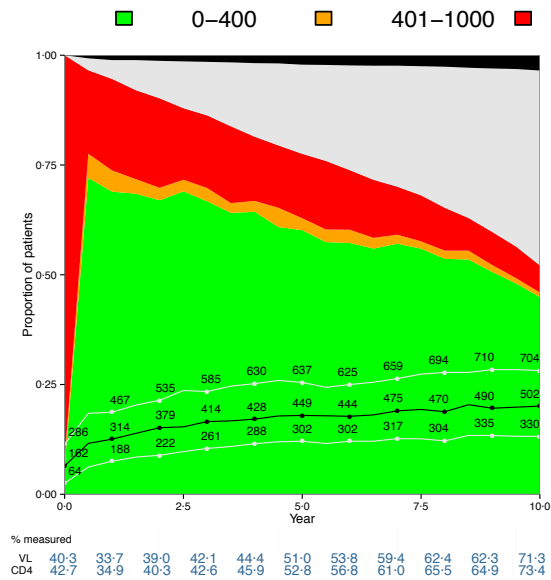

(a) HF/CMH-Argentina

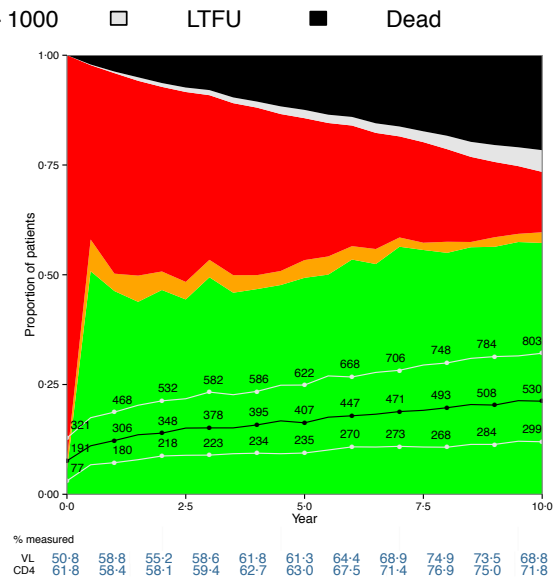

(b) INI-Brazil

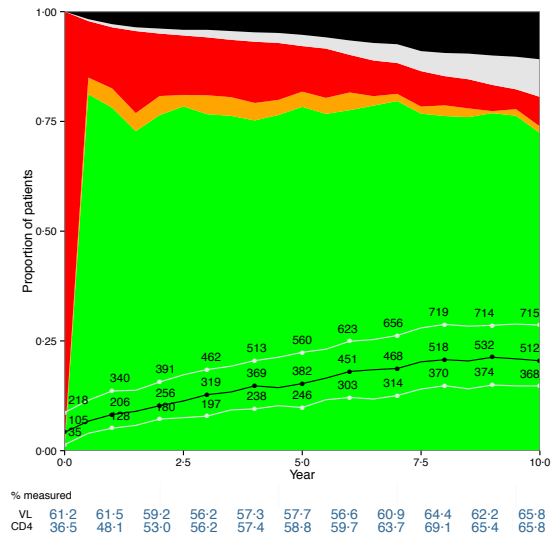

(c) FA-Chile

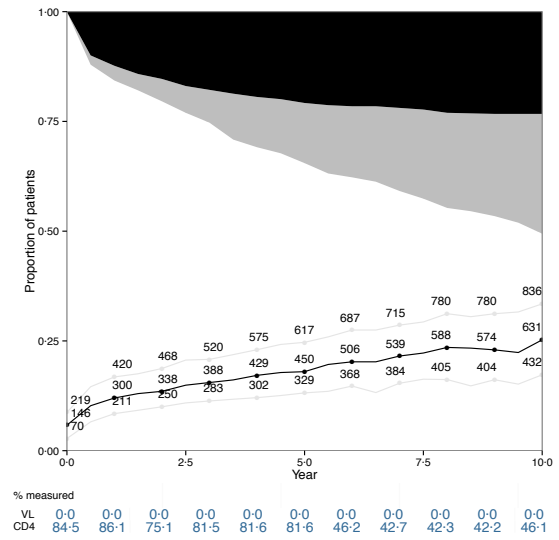

(d) GHESKIO-Haiti

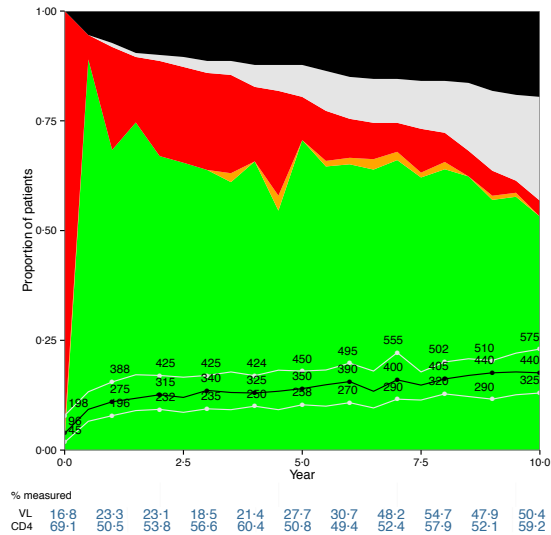

(e) IHSS/HE-Honduras

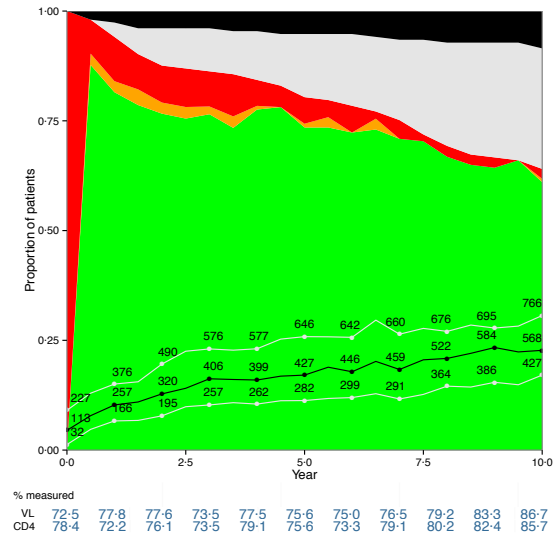

(f) INCMNSZ-Mexico

Supplement: S1 Fig — Median (black line) as well as 25th percentile and 75th percentile CD4 (white lines) are also displayed. The percentage of active patients with a measured viral load and CD4 is given at the bottom of the figure. (PDF) [file pone.0179769.s001.pdf]
