# Supplementary material for: A decade of HAART in Latin America: Long term outcomes among the first wave of HIV patients to receive combination therapy
Source: PLoS One. 2017 Jun 26;12(6):e0179769. doi: 10.1371/journal.pone.0179769 (PMC5484471; doi:10.1371/journal.pone.0179769)
Supplement: S2 Fig — Patients were classified as lost to follow-up after their last documented visit date, regardless of the timing of the previous regimen dispensation. (PDF) [file pone.0179769.s002.pdf]

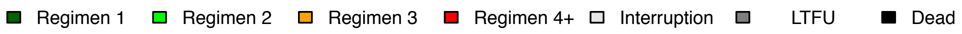

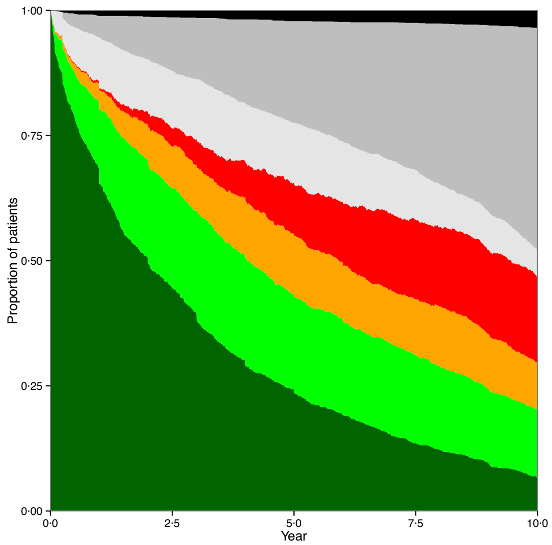

(a) HF/CMH-Argentina

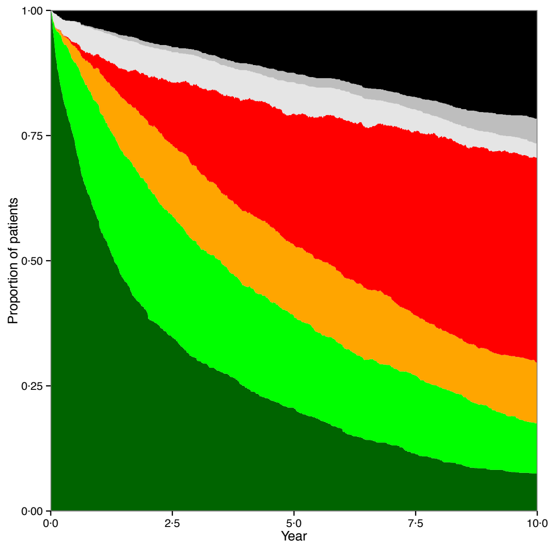

(b) INI-Brazil

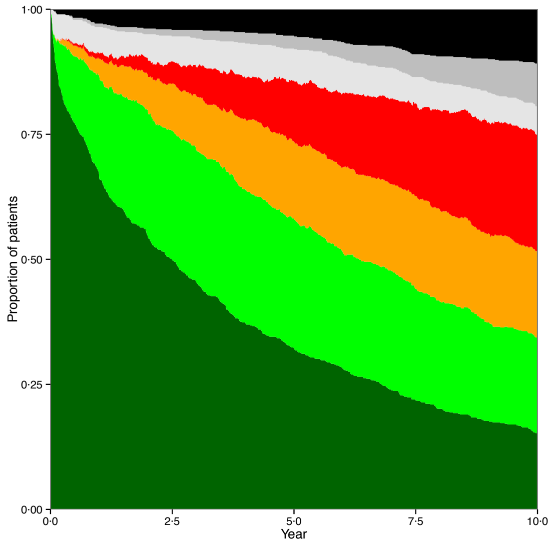

(c) FA-Chile

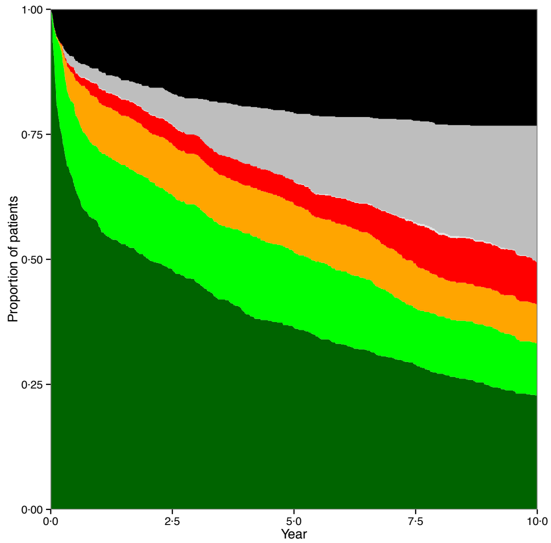

(d) GHESKIO-Haiti

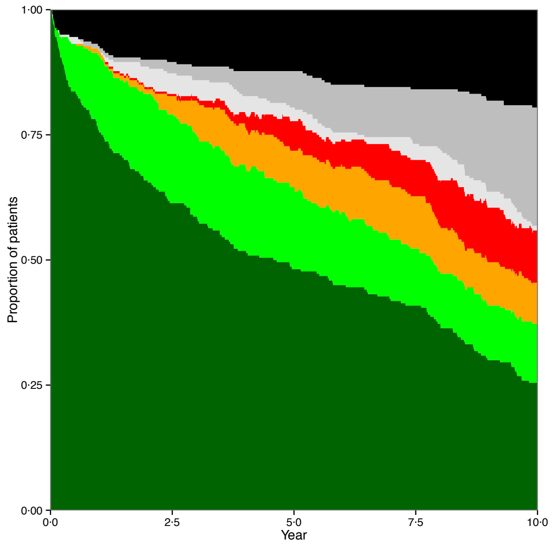

(e) IHSS/HE-Honduras

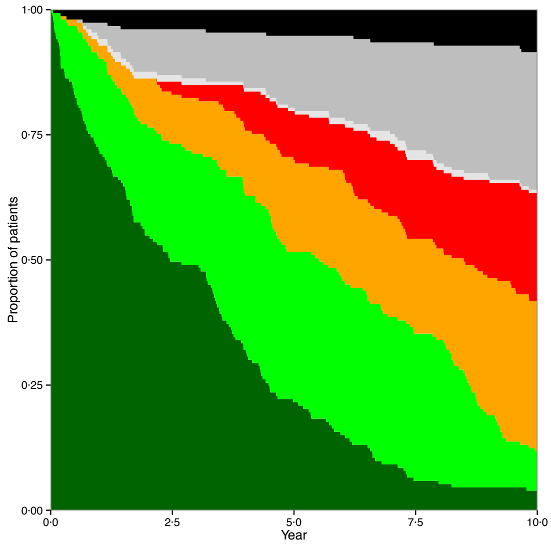

(f) INCMNSZ-Mexico
