# Supplementary material for: A decade of HAART in Latin America: Long term outcomes among the first wave of HIV patients to receive combination therapy
Source: PLoS One. 2017 Jun 26;12(6):e0179769. doi: 10.1371/journal.pone.0179769 (PMC5484471; doi:10.1371/journal.pone.0179769)
Supplement: S1 Table — (PDF) [file pone.0179769.s003.pdf]

	HF/CMH-Argentina n=549	INI-Brazil n=775	FA-Chile n=416	IHSS/HE-Honduras n=47	INCMNSZ-Mexico n=112	Combined n=1899
	Value	Value	Value	Value	Value	
Age, years	34 (29 - 40)	36 (31 - 43)	36 (31 - 43)	35 (30 - 39.5)	34 (27 - 40)	35 (30 - 42)
Sex						
Female	155(28%)	295(38%)	57(14%)	26(55%)	20(18%)	553(29%)
Male	394(72%)	480(62%)	359(86%)	21(45%)	92(82%)	1346(71%)
Probable Route of Infection						
Heterosexual	183(33%)	389(50%)	110(26%)	27(57%)	39(35%)	748(39%)
Homosexual or Bisexual	158(29%)	258(33%)	300(72%)	2(4%)	69(62%)	787(41%)
IDU	44(8%)	21(3%)	3(1%)	1(2%)	3(3%)	72(4%)
Other	4(1%)	24(3%)	3(1%)	0(0%)	1(1%)	32(2%)
Unknown	160(29%)	83(11%)	0(0%)	17(36%)	0(0%)	260(14%)
Clinical Stage						
AIDS	139(25%)	47(6%)	129(31%)	24(51%)	60(54%)	399(21%)
not AIDS	160(29%)	530(68%)	142(34%)	22(47%)	41(37%)	895(47%)
Missing	250(46%)	198(26%)	145(35%)	1(2%)	11(10%)	605(32%)
Nadir CD4, cells/mm^3	139 (53 - 246)	174 (76 - 280)	144 (48 - 228)	116 (40 -214	106 (36 - 213	152 (63 - 260
Missing	162(30%)	117(15%)	197(47%)	15(32%)	22(20%)	513(27%)
Baseline CD4, cells/mm^3	147 (58 - 268)	207 (89 - 341)	144 (45 - 220)	116 (42 - 215)	114 (36 - 226)	166 (66 - 287)
Missing	183(33%)	187(24%)	248(60%)	17(36%)	24(21%)	659(35%)
Baseline VL ($\log_{10}$)	5.0 (4.4 - 5.5)	4.7 (4.0 - 5.3)	5.0 (4.4 - 5.5)	5.0 (4.6 - 5.1)	4.9 (4.8 - 4.9)	4.9 (4.3 - 5.4)
Baseline VL (undetectable)						
Yes	23(4%)	25(3%)	4(1%)	1(2%)	0(0%)	53(3%)
No	323(59%)	449(58%)	293(70%)	16(34%)	82(73%)	1163(61%)
Missing	203(37%)	301(39%)	119(29%)	30(64%)	30(27%)	683(36%)
Initial Regimen Class						
NNRTI	255(46%)	289(37%)	280(67%)	44(94%)	80(71%)	948(50%)
Boosted PI	145(26%)	58(7%)	5(1%)	0(0%)	19(17%)	227(12%)
Unboosted PI	101(18%)	405(52%)	119(29%)	3(6%)	10(9%)	638(34%)
3 NRTI	42(8%)	10(1%)	5(1%)	0(0%)	3(3%)	60(3%)
Other	6(1%)	13(2%)	7(2%)	0(0%)	0(0%)	26(1%)
Missing	0(0%)	0(0%)	0(0%)	0(0%)	0(0%)	0(0%)
Year of Initial Regimen						
1996	3(1%)	40(5%)	0(0%)	0(0%)	0(0%)	43(2%)
1997	10(2%)	138(18%)	6(1%)	0(0%)	0(0%)	154(8%)
1998	21(4%)	110(14%)	18(4%)	0(0%)	0(0%)	149(8%)
1999	40(7%)	94(12%)	85(20%)	0(0%)	0(0%)	219(12%)
2000	100(18%)	111(14%)	47(11%)	0(0%)	0(0%)	258(14%)
2001	118(21%)	139(18%)	59(14%)	2(4%)	3(3%)	321(17%)
2002	114(21%)	64(8%)	124(30%)	6(13%)	42(38%)	350(18%)
2003	143(26%)	79(10%)	77(19%)	39(83%)	67(60%)	405(21%)
ART naive						
Yes	335(61%)	259(33%)	343(82%)	44(94%)	111(99%)	1092(58%)
No	214(39%)	516(67%)	73(18%)	3(6%)	1(1%)	807(42%)
Unconfirmed	(%)	(%)	(%)	(%)	(%)	(%)
