# Supplementary material for: A decade of HAART in Latin America: Long term outcomes among the first wave of HIV patients to receive combination therapy
Source: PLoS One. 2017 Jun 26;12(6):e0179769. doi: 10.1371/journal.pone.0179769 (PMC5484471; doi:10.1371/journal.pone.0179769)
Supplement: S2 Table — (PDF) [file pone.0179769.s004.pdf]

	HF/CMH-Argentina n=1082	INI-Brazil n=543	FA-Chile n=260	IHSS/HE-Honduras n=155	INCMNSZ-Mexico n=32	Combined n=2072
	Value	Value	Value	Value	Value	
Age, years	33 (28 - 39)	35 (30 - 41)	35 (29.75 - 40.25)	34 (28.5 - 39)	33 (30 - 40.5)	34 (29 - 40)
Sex						
Female	306(28%)	177(33%)	48(18%)	81(52%)	6(19%)	618(30%)
Male	776(72%)	366(67%)	212(82%)	74(48%)	26(81%)	1454(70%)
Probable Route of Infection						
Heterosexual	341(32%)	221(41%)	88(34%)	83(54%)	13(41%)	746(36%)
Homosexual or Bisexual	227(21%)	193(36%)	166(64%)	4(3%)	19(59%)	609(29%)
IDU	116(11%)	16(3%)	2(1%)	0(0%)	0(0%)	134(6%)
Other	10(1%)	13(2%)	3(1%)	1(1%)	0(0%)	27(1%)
Unknown	388(36%)	100(18%)	1(0%)	67(43%)	0(0%)	556(27%)
Clinical Stage						
AIDS	173(16%)	25(5%)	63(24%)	81(52%)	13(41%)	355(17%)
not AIDS	147(14%)	205(38%)	59(23%)	67(43%)	8(25%)	486(23%)
Missing	762(70%)	313(58%)	138(53%)	7(5%)	11(34%)	1231(59%)
Nadir CD4, cells/mm^3	155 (57 - 286)	168 (59 - 266)	159 (42 - 268)	112 (55 -196	162 (30 - 236	151 (55 - 262
Missing	744(69%)	244(45%)	165(63%)	31(20%)	7(22%)	1191(57%)
Baseline CD4, cells/mm^3	149 (55 - 280)	183 (76 - 288)	117 (38 - 230)	96 (52 - 197)	162 (30 - 236)	150 (55 - 264)
Missing	785(73%)	309(57%)	186(72%)	40(26%)	7(22%)	1327(64%)
Baseline VL ($\log_{10}$)	5.0 (4.5 - 5.5)	4.8 (4.1 - 5.4)	4.9 (4.4 - 5.4)	5.0 (4.7 - 5.0)	4.9 (4.7 - 4.9)	4.9 (4.4 - 5.4)
Baseline VL (undetectable)						
Yes	19(2%)	11(2%)	1(0%)	0(0%)	0(0%)	31(1%)
No	266(25%)	189(35%)	117(45%)	17(11%)	24(75%)	613(30%)
Missing	797(74%)	343(63%)	142(55%)	138(89%)	8(25%)	1428(69%)
Initial Regimen Class						
NNRTI	434(40%)	174(32%)	166(64%)	141(91%)	21(66%)	936(45%)
Boosted PI	161(15%)	56(10%)	15(6%)	1(1%)	4(12%)	237(11%)
Other	11(1%)	15(3%)	7(3%)	0(0%)	0(0%)	33(2%)
Missing	0(0%)	0(0%)	0(0%)	0(0%)	0(0%)	0(0%)
Year of Initial Regimen						
1996	51(5%)	69(13%)	4(2%)	1(1%)	0(0%)	125(6%)
1997	131(12%)	91(17%)	3(1%)	2(1%)	0(0%)	227(11%)
1998	159(15%)	91(17%)	5(2%)	2(1%)	0(0%)	257(12%)
1999	146(13%)	60(11%)	29(11%)	1(1%)	0(0%)	236(11%)
2000	150(14%)	67(12%)	38(15%)	1(1%)	0(0%)	256(12%)
2001	190(18%)	54(10%)	59(23%)	4(3%)	2(6%)	309(15%)
2002	129(12%)	71(13%)	68(26%)	32(21%)	17(53%)	317(15%)
2003	126(12%)	40(7%)	54(21%)	112(72%)	13(41%)	345(17%)
ART naive						
Yes	335(31%)	108(20%)	157(60%)	147(95%)	31(97%)	778(38%)
No	746(69%)	435(80%)	103(40%)	8(5%)	1(3%)	1293(62%)
Unconfirmed	1(0%)	0(0%)	0(0%)	0(0%)	0(0%)	1(0%)
