# Supplementary material for: A decade of HAART in Latin America: Long term outcomes among the first wave of HIV patients to receive combination therapy
Source: PLoS One. 2017 Jun 26;12(6):e0179769. doi: 10.1371/journal.pone.0179769 (PMC5484471; doi:10.1371/journal.pone.0179769)
Supplement: S3 Table — P-values for continuous variables were calculated using a Wilcoxon Rank Sum test. P-values for categorical variables were calculated using a Chi-squared test. (PDF) [file pone.0179769.s005.pdf]

	HF/CMH-Argentina n=1631	INI-Brazil n=1318	FA-Chile n=676	IHSS/HE-Honduras n=202	INCMNSZ-Mexico n=144	Combined n=3971
Age, years	0.078	0.001	0.007	0.557	0.876	< 0.001
Sex	1.000	0.048	0.120	0.840	1.000	0.651
Female						
Male						
Probable Route of Infection	0.002	< 0.001	0.163	0.346	0.707	< 0.001
Heterosexual						
Homosexual or Bisexual						
IDU						
Other						
Unknown						
Clinical Stage	0.071	0.276	0.527	0.893	1.000	< 0.001
AIDS						
not AIDS						
Missing						
Nadir CD4, cells/mm^3	0.084	0.177	0.608	0.794	0.717	0.854
Missing	< 0.001	< 0.001	< 0.001	0.132	0.978	< 0.001
Baseline CD4, cells/mm^3	0.678	0.032	0.942	0.975	0.846	0.024
Missing	< 0.001	< 0.001	0.002	0.231	1.000	< 0.001
Baseline VL ($\log_{10}$)	0.846	0.135	0.692	0.890	0.705	0.123
Baseline VL (undetectable)	1.000	1.000	1.000	1.000	NA	0.740
Yes						
No						
Missing						
Initial Regimen Class	< 0.001	0.055	0.003	0.868	0.491	0.014
NNRTI						
Boosted PI						
Other						
Missing						
Year of Initial Regimen	< 0.001	< 0.001	< 0.001	0.756	0.132	< 0.001
1996						
1997						
1998						
1999						
2000						
2001						
2002						
2003						
ART naive	< 0.001	< 0.001	< 0.001	1.000	0.924	< 0.001
Yes						
No						
Unconfirmed						
